# Outcomes of aortic valvuloplasty with pericardium patch for congenital aortic stenosis and regurgitation in pediatric patients

**DOI:** 10.3389/fcvm.2025.1724329

**Published:** 2026-01-21

**Authors:** Pinyan Huang, Junjie Zong, Weicong Ye, Song Wang, Ran Li, Han Zhang, Zilong Luo, Jiahong Xia, Jizhang Yu, Jie Wu, Cheng Zhou

**Affiliations:** 1 Department of Cardiovascular Surgery, Union Hospital, Tongji Medical College, Huazhong University of Science and Technology, Wuhan, China; 2 Key Laboratory of Organ Transplantation, Ministry of Education, Chinese Academy of Medical Sciences, Wuhan, China; 3 Center for Translational Medicine, Union Hospital, Tongji Medical College, Huazhong University of Science and Technology, Wuhan, China; 4 Institute of Translational Medicine, Tongji Medical College, Huazhong University of Science and Technology, Wuhan, China

**Keywords:** aortic valvuloplasty, autologous pericardial patch, congenital aortic stenosis, congenital regurgitation, pediatric cardiac surgery

## Abstract

**Background:**

Surgical strategies for congenital aortic stenosis and regurgitation in children, particularly in infants (<1 year), remain controversial. Aortic valvuloplasty (AVP) with pericardial patch has gained increasing attention, but its durability and clinical benefits remain uncertain.

**Methods:**

We retrospectively analyzed pediatric patients (≤12 years) undergoing AVP with pericardial patch in our center between July 2017 and July 2025. Infants (<1 year) were analyzed separately as subgroups. Primary outcome was the change in aortic valve hemodynamics, including median peak gradient, median peak velocity, and degree of aortic regurgitation. Secondary outcomes included major complications, overall survival, and freedom from reoperation.

**Results:**

A total of 35 patients were included, with a median age of 2 years. Among them, 17 were infants, with a median age of 2 months. The median peak aortic valve gradient decreased from 67.0 mmHg to 33.0 mmHg (*p* < 0.001), and the median peak velocity decreased from 4.1 m/s to 2.9 m/s (*p* < 0.001), postoperatively. No new moderate or severe aortic regurgitation was observed early postoperatively, and preexisting lesions of this severity were resolved. There were no in-hospital deaths or severe complications. At four years, survival was 96% and freedom from reoperation 75.4% in the overall cohort; in infants, survival was 100% with 66.7% freedom from reoperation.

**Conclusions:**

AVP with pericardium patch is a safe and effective procedure for congenital aortic stenosis and regurgitation in pediatric patients. It represents a promising surgical option for pediatric patients, including infants.

## Graphical Abstract

**Figure:**
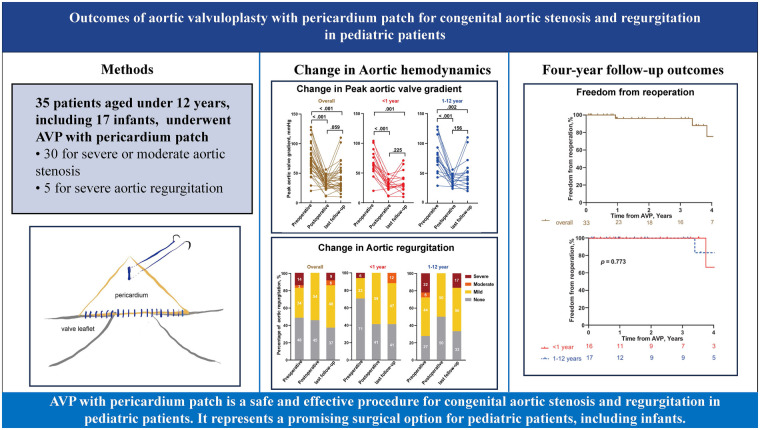

## 1 Introduction

The optimal surgical technique for congenital aortic valve defects remains one of the most debated topics in pediatric cardiac surgery. Common surgical options for the management of congenital aortic stenosis include the Ross procedure, aortic valve replacement (AVR), and aortic valvuloplasty (AVP). Although AVR effectively alleviates stenosis, its use in pediatric patients is limited by material constraints, substantial mortality especially in the very young, and considerable reintervention hazards as children grow (1). The Ross procedure is favored for severe left ventricular outflow tract obstruction or failed valvuloplasty. However, its complexity and risks, particularly in infants, restrict its application (2, 3). Balloon aortic valvuloplasty (BAV) or surgical aortic commissurotomy, which involve opening up stenotic valves, are recommended for effectively alleviating the stenosis but are frequently associated with a high risk of significant postoperative aortic regurgitation. Against this background, neonates and infants with congenital aortic stenosis constitute a particularly high-risk subgroup. In this age range, aortic annuli are very small, leaflet tissue is fragile, and surgical exposure is limited, while rapid somatic growth increases the lifetime risk of reinterventions. As a result, the balance between immediate relief of stenosis and preservation of native valve structures is especially delicate, and both early mortality and long-term durability remain major concerns for any operative strategy in this population.

To address these limitations, AVP with patch reconstruction has emerged as a promising option for severe congenital aortic defects (4–6). This technique focuses on restoring normal valve geometry through cusp and root reconstruction, with minimal invasion of the physiological structure of the aortic valve. However, the specific technical details of patch reconstruction remain underdeveloped, and its application in infants is still rare and debated. Therefore, further attempts are needed to validate the efficacy and safety of AVP with pericardium patch.

The AVP involving with commissures reconstruction using a triangular pericardium patch was initiated at our center in 2017. We retrospectively analyzed the in-hospital and four-year follow-up outcomes of patients aged under 12 years, and predefined infants (≤1 year) as a key subgroup of particular clinical interest. This study aimed to evaluate its safety, efficacy, and potential as an option for congenital aortic stenosis.

## 2 Patients and methods

### 2.1 Ethics statement

The study was approved by the Ethics Committee of Union Hospital, Tongji Medical College, Huazhong University of Science and Technology [(2023) 0848-01]. The requirement for individual patient consent was formally waived by the ethics committee due to the retrospective nature of the study. All information was anonymized and handled in strict accordance with the information security protocols of Wuhan Union Hospital.

### 2.2 Patient population

Data of consecutive pediatric patients aged under 12 years who underwent AVP with pericardium patch for aortic stenosis and regurgitation between July 2017 and July 2025 were included. Primary outcome was the change in aortic valve hemodynamics, including median peak gradient, median peak velocity, and degree of aortic regurgitation. Secondary outcomes included major complications, overall survival, and freedom from reoperation. Data collection was based on electronic medical records.

In our institution, for children with congenital aortic valve disease, AVP with a pericardial patch is preferentially chosen when the valve is considered repairable. Specifically, AVP was offered when (i) at least moderate aortic stenosis and/or regurgitation was documented on transthoracic echocardiography, (ii) the aortic annulus was of adequate size for body surface area without severe hypoplasia, and (iii) valve morphology was deemed amenable to repair by the surgical team (for example, no extensive leaflet calcification or severely dysplastic cusps precluding reconstruction). Children with complex multivalve disease, severe annular hypoplasia or associated lesions requiring aortic root replacement were treated with prosthetic aortic valve replacement (AVR) instead. If an initially attempted AVP did not achieve satisfactory valve function and no further repair was feasible, the operation was converted to AVR; such patients were analyzed as part of the replacement group and were not included in the present AVP cohort.

Given the specific anatomical and physiological challenges of neonates and infants with congenital aortic valve disease, infants (<1 year at the time of surgery) were prespecified as a key subgroup of interest. The study was designed to compare early and mid-term outcomes between infants and older children in order to explore the feasibility and durability of this AVP technique in the most vulnerable age group.

Aortic valve lesions were graded by comprehensive transthoracic echocardiogram (TTE) according to current pediatric practice. Aortic stenosis was assessed using Doppler-derived peak transvalvular velocity and peak gradient and was categorized as mild (peak velocity ≤3.0 m/s), moderate (3.0–4.0 m/s) or severe (≥4.0 m/s). Aortic regurgitation was evaluated using an integrated approach based on color Doppler jet characteristics (jet width and vena contracta), and was classified as none, mild, moderate or severe. Based on the predominant hemodynamic lesion, aortic valve defects were classified into three categories. Predominant aortic stenosis was defined as at least moderate aortic stenosis with none or mild aortic regurgitation; predominant aortic regurgitation as at least moderate aortic regurgitation with none or mild aortic stenosis; and mixed disease as the presence of at least moderate aortic stenosis and at least moderate aortic regurgitation in the same patient. These echocardiographic criteria were used both for preoperative lesion grading and for postoperative and follow-up assessment.

### 2.3 Surgical techniques

The operative technique was similar to previously described methods (6, 7). First of all, CPB was established with an aortic cannula in the ascending aorta and venous cannulae in the superior and inferior vena cavae via the right atrium. The ascending aorta was then clamped, followed by a controlled incision at the root, and antegrade cardioplegia was delivered through the coronary ostia for cardiac arrest. After this, pathological changes were assessed through the incision at the ascending aortic root, and fused commissures were incised, with thickened leaflets and plaques excised as needed.

**Figure 1:**
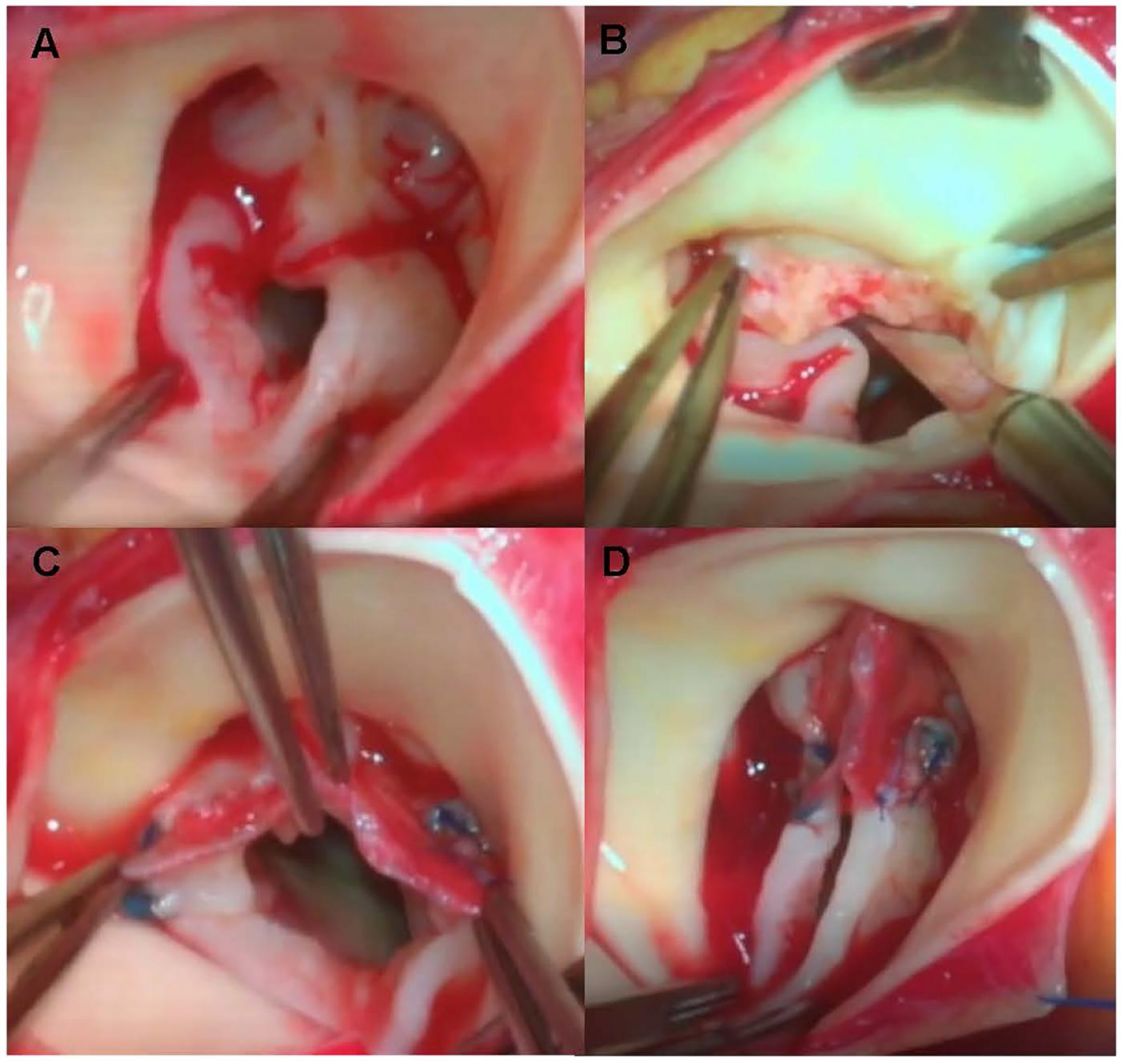
Intraoperative views of aortic valvuloplasty with an autologous pericardial patch. **(A)** Intraoperative view of the congenital aortic valve after aortotomy, showing commissural fusion and leaflet thickening before repair. **(B)** After longitudinal commissurotomy of the fused commissure and careful thinning of the thickened leaflet tissue. **(C)** Completion of suturing of the basal edge of the glutaraldehyde-treated autologous pericardial triangular patch to the annulus and adjacent leaflet edges along the reconstructed commissure. **(D)** Final appearance of the reconstructed valve after suspension of the apical tip of the triangular patch with a single central stitch, demonstrating restored commissural height and central leaflet coaptation before aortic closure.

A glutaraldehyde-treated autologous pericardial patch was used for commissures reconstruction. Autologous pericardial tissue was obtained from the posterior pericardial wall and fixed in 0.6% glutaraldehyde solution at room temperature for 10 min, followed by three rinses in physiologic saline, which was adapted from previously published protocols (3, 8, 9). Before trimming the pericardium into a triangular patch, the surgeon carefully measured the commissural defect with a caliper. The patch was then trimmed into a triangular shape, with the base measuring approximately twice the length of the incised raphe and the height slightly exceeding the sinotubular ridge. The patch was secured in two steps. First, the base was sewn horizontally to the commissure defect and extended to the adjacent leaflet, consistent with conventional methods. We would use this patch to repair localized leaflet deficiency adjacent for repairing commissure when needed. Then, the apex of the triangular patch was suspended to the aortic wall to a level above the sinotubular junction with a single suture, effectively reconstructing the new commissure with reduced complexity. Finally, valve leaflet motion was assessed using a dilator to confirm sufficient opening without redundancy or excessive tension. No clinically evident patch dehiscence or patch-related structural failure was observed during follow-up in this series. Intraoperative hemodynamic assessment is performed using transesophageal echocardiography (TEE) after completion of the repair, mainly to confirm that postoperative peak transvalvular velocity of approximately ≤3.0 m/s, aortic regurgitation is mild or absent, and left ventricular systolic function remains normal. If intraoperative assessment suggested that the patch reconstruction was unsatisfactory, the repair was revised and, when adequate valve function could not be achieved, the procedure was converted to aortic valve replacement. Once satisfactory function was verified, the aortic incision was closed using a continuous suture. The key steps of the triangular pericardial patch reconstruction are illustrated in Figure 1.

During the study period, all procedures were performed by one senior surgeon. The decision to perform the procedure was made during the preoperative discussion and finalized based on intraoperative assessment.

### 2.4 Postoperative care

A first postoperative TTE was routinely performed 3–4 days after surgery, before hospital discharge, and was defined as the early postoperative hemodynamic assessment. Following AVP, all patients received low-dose aspirin (3–5 mg/kg/day, up to a maximum of 100 mg) for 6 months. Antiplatelet therapy was then discontinued in the absence of other indications for antithrombotic treatment. Post-resuscitation circulatory support time was defined as the duration of postoperative hemodynamic support with continuous inotropic and/or vasoactive infusions, measured from admission to the intensive care unit after surgery until complete discontinuation of all pharmacologic circulatory support, and was recorded in minutes.

### 2.5 Follow-up

After discharge, patients were followed at our outpatient clinic or at their referring hospitals according to routine clinical practice. During the follow-up period, TTE were scheduled at approximately 3 months postoperatively and every 6 months thereafter. Clinical and imaging data for this study were retrieved retrospectively from the institutional electronic medical record and outpatient databases and, when necessary, were supplemented by a one-time telephone follow-up conducted under ethics committee approval to ascertain vital status and aortic valve reinterventions. All chart-based and telephone follow-up information included in this report was obtained before the database was locked on 31 July 2025, prior to conducting the analyses.

### 2.6 Statistical analysis

Continuous variables are expressed as mean ± standard deviation for normally distributed data or as median with interquartile range (IQR) for non-normally distributed data, as determined by the Shapiro–Wilk test. Between-group comparisons were performed using the Student *t*-test or the Mann–Whitney *U*-test, as appropriate. Categorical variables are reported as frequencies and percentages and were compared using the chi-square test or Fisher's exact test, depending on data distribution. For baseline clinical characteristics and perioperative outcomes, BMI and BSA were calculated in 30 patients with non-missing height/length and were analyzed using an available-case approach, while other values were analyzed in the entire cohort of 35 patients with completed data.

For echocardiographic and hemodynamic analyses, follow-up transthoracic echocardiographic (TTE) data were available in 33 patients. Thus, echocardiographic parameters were compared across preoperative, postoperative, and last follow-up time points in 33 patients with complete imaging data. For all analyses, “preoperative” values refer to the last TTE before surgery, “postoperative” values refer to the early in-hospital TTE at 3–4 days, and “last follow-up” values refer to the most recent TTE available for each patient within this surveillance schedule. For each two-time-point comparison, only patients with measurements at both time points were included. Given the small sample size and the exploratory, single-center nature of this study, no formal adjustment for multiple comparisons was applied. Paired comparisons across different time points were performed using the Wilcoxon signed-rank test. Survival and freedom from reoperation were analyzed using the Kaplan–Meier method and between-group comparisons were performed using log-rank test. Right censoring was done at last follow-up, death, or reoperation. All statistical analyses were conducted using GraphPad Prism version 10.0.0 for Windows (GraphPad Software, Boston, MA, USA).

## 3 Results

### 3.1 Baseline characteristics

A total of 35 pediatric patients, including 17 infants, underwent AVP. Predominant aortic stenosis was present in 80% of patients, predominant aortic regurgitation in 9%, and mixed aortic stenosis/aortic regurgitation in 11%. Baseline characteristics of patients were presented in Table 1. The median age of the entire cohort was 2.0 years (IQR 0.2–7.5), and 20% were female. Median body weight was 11.0 kg overall, 5.0 kg in infants, and 28.8 kg in older children. Correspondingly, the median body surface area was 0.6 m^2^ in all patients and 0.3 m^2^ among infants (*p* < 0.001). The median body mass index was 16.1 kg/m^2^, without a significant difference between age groups (*p* = 0.471). Severe or moderate aortic stenosis was the primary lesion in 30 patients (86%), whereas 5 (14%) had severe aortic regurgitation as the main indication. Among infants, 94% had aortic stenosis and 6% had aortic regurgitation; in older children, 78% and 22%, respectively.

**Table 1:** Patients' baseline characteristics.

Variable	Overall (*n* = 35)	Age ≤1 year (*n* = 17)	Age >1 year (*n* = 18)	*p*-value
Age	2.0 (0.2, 7.5) years	2.0 (1.0, 5.0) months	7.5 (3.9, 10.0) years	<0.001
Female	7 (20)	3 (18)	4 (22)	>0.999
Weight, kg	11.0 (5.5, 28.8)	5.0 (3.5, 6.9)	28.8 (16.1, 36.5)	<0.001
BMI, kg/m^2^	16.1 (14.2, 19.8)	16.5 (13.7, 18.7)	15.7 (14.3, 20.6)	0.471
BSA, m^2^	0.6 (0.3, 1.1)	0.3 (0.2, 0.3)	1.0 (0.7, 1.2)	<0.001
Aortic valve defects				0.08
Predominant AS	28 (80)	16 (94)	12 (67)	
Mixed AS/AR	4 (11)	0 (0)	4 (22)	
Predominant AR	3 (9)	1 (6)	2 (11)	
Concomitant surgery				0.153
Valvuloplasty alone	24 (69)	9 (53)	15 (83)	
Mitral valve repair	3 (9)	3 (18)	0 (0)	
Ventricular septal defect	2 (6)	1 (6)	1 (6)	
Patent ductus arteriosus	6 (16)	4 (23)	2 (11)	
Previous cardiac valve surgery				>0.999
None	34 (97)	17 (100)	17 (94)	
Surgical aortic valvuloplasty	1 (3)	0 (0)	1 (6)	

Median (IQR) for continuous variables and number (percentage) for categorical variables. BMI and BSA were available in 30 patients, whereas all other variables were available in all 35 patients. AR, aortic regurgitation; BMI, body mass index; BSA, body surface area; AS, aortic stenosis.

### 3.2 Intraoperative data

Median operative time was 155 min (IQR 139–206) overall and 170 min (IQR 150–210) among infants, compared with 150 min (IQR 135–180) in older children (*p* = 0.174). The median cardiopulmonary bypass (CPB) time was 66.0 min (63.0–77.0) overall and 75.0 min (56.5–89.0) in infants (*p* = 0.270). Aortic cross-clamp time averaged 38.0 min (29.0–48.5), similar between groups (*p* = 0.595). The post-resuscitation circulatory support time was longer in infants (23.0 min) than in older children (15.0 min, *p* = 0.005). Although the infant group had longer operative and bypass times compared to patients older than 1 year, the differences were not statistically significant. Intraoperative blood transfusion was required in 9 patients (26%), with comparable rates between infants (24%) and older children (28%) (*p* > 0.999). No intraoperative mortality occurred in any group. All patients were weaned successfully from CPB, and no conversion to valve replacement was necessary (Table 2).

**Table 2:** Patients' intraoperative data.

Variable	Overall (*n* = 35)	Age ≤1 year (*n* = 17)	Age >1 year (*n* = 18)	*p*-value
Operative time, min	155 (139, 206)	170 (150, 210)	150 (135, 180)	0.174
CPB time, min	66.0 (63.0, 77.0)	75.0 (56.5, 89.0)	64.0 (63.0, 71.8)	0.270
Aortic cross-clamp time, min	38.0 (29.0, 48.5)	35.5 (28.0, 50.8)	39.0 (34.0, 47.0)	0.595
Post-resuscitation circulatory support time, min	19.5 (14.0, 23.0)	23.0 (18.0, 31.0)	15.0 (13.0, 21.0)	0.005
Intraoperative blood transfusion	9 (26)	4 (24)	5 (28)	>0.999
Intraoperative mortality	0 (0)	0 (0)	0 (0)	>0.999

Median (IQR) for continuous variables and number (percentage) for categorical variables. Comparisons were performed based on 35 patients with complete data. AVP, aortic valvuloplasty; CPB, cardiopulmonary bypass; NNIS, National Nosocomial Infection Surveillance.

### 3.3 Postoperative data and follow-up outcomes

There were no deaths or other major complications during the hospitalization period, including severe bleeding, stroke, myocardial infarction, atrial fibrillation, complete heart block, sepsis, deep sternal infection, chronic renal failure and other early postoperative adverse events defined by the European Association for Cardio-Thoracic Surgery Mortality (10). The median ICU stay was 3 days (IQR 2–7), significantly longer in infants (7 days [IQR 5–9]) than in older children (2 days [IQR 2–2], *p* < 0.001). The median total hospital stay was 22 days (IQR 17–26), again longer in infants [26 days (IQR 18–34)] than in older children [18 days (IQR 16–24), *p* = 0.037] (Table 3).

**Table 3:** Patients' postoperative outcomes and follow-up outcomes.

Variable	Overall (*n* = 35)	Age ≤1 year (*n* = 17)	Age >1 year (*n* = 18)	*p*-value
Death during the hospitalization period	0 (0)	0 (0)	0 (0)	>0.999
Pneumonia	2 (6)	2 (12)	0 (0)	0.228
Tracheostomy or reintubation	0 (0)	0 (0)	0 (0)	>0.999
Lactate level on postoperative day 1, mmol/L	1.4 (0.9, 1.9)	1.6 (1.2, 2.1)	1.2 (0.9, 1.7)	0.244
ICU length of stay, day	3 (2, 7)	7 (5, 9)	2 (2, 2)	<0.001
Hospital length of stay, day	22 (17, 26)	26 (18, 34)	18 (16, 24)	0.037
Death during follow-up period	1 (5)	0 (0)	1 (6)	>0.999
Type of reoperation	5 (14)	2 (12)	3 (17)	>0.999
Ross procedure	2 (40)	1 (50)	1 (33)	
Mechanical heart valve replacement	3 (60)	1 (50)	2 (67)	

Median (IQR) for continuous variables and number (percentage) for categorical variables. Comparisons of postoperative outcomes were performed in 35 patients with complete data. Analyses of follow-up outcomes were performed in 33 patients with available follow-up data. ICU, intensive care unit.

**Figure 2:**
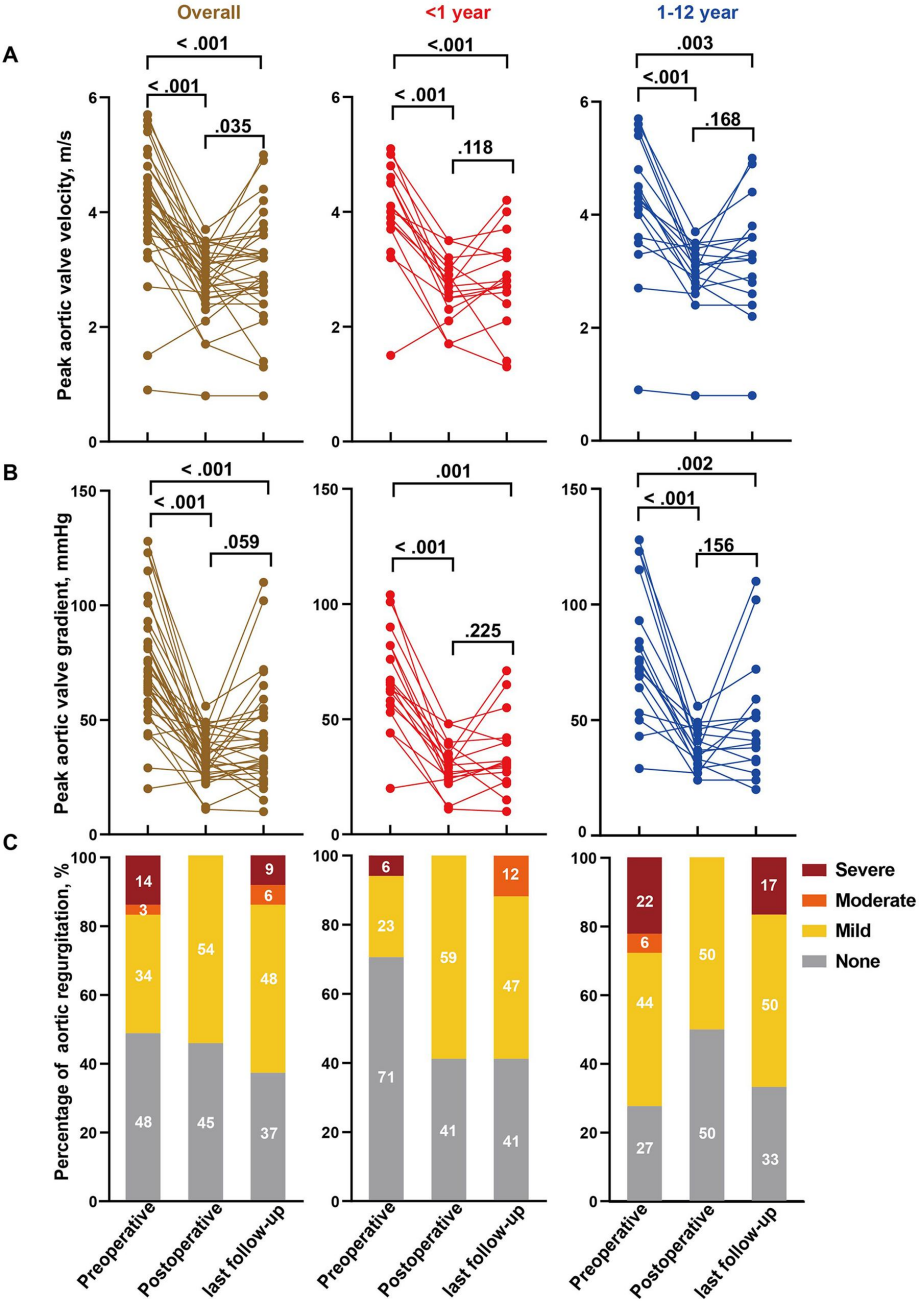
Hemodynamic alterations of the aortic valve in patients underwent AVP with a pericardium patch. Comparison of preoperative, postoperative and last follow-up aortic valve peak velocity **(A)**, peak gradient **(B)** and regurgitation **(C)** in overall cohort, infants (≤1 year) and children age >1 year. Analyses were performed based on 33 patients with follow-up data. Percentages may not sum to 100% because of rounding.

Follow-up data were available for 33 patients. Early postoperative TTE confirmed satisfactory valve competence after repair in this cohort, with peak transvalvular velocity <3.0 m/s and no more than mild aortic regurgitation in the great majority of patients. Compared with preoperative values, early postoperative TTE findings demonstrated marked hemodynamic improvement: the median peak aortic valve velocity decreased from 4.1 (3.9–4.5) m/s to 2.9 (2.7–3.1) m/s (*p* < 0.001), and the median peak gradient decreased from 67.0 (60.0–81.5) mmHg to 33.0 (28.5–38.5) mmHg (*p* < 0.001). Comparable reductions were observed in infants, with median velocity falling from 4.0 to 2.8 m/s and peak gradient from 65.0 to 32.0 mmHg (*p* = 0.015). Pre-existing moderate or severe aortic regurgitation was completely corrected, and no new moderate or severe regurgitation was detected at the early postoperative assessment (Figure 2, Supplementary Table S1). The median clinical and echocardiographic follow-up duration was 2.5 years (IQR 1.0–4.1 years). At the last follow-up, mean aortic valve velocity (*p* = 0.035) and gradient (*p* = 0.059) increased compared with postoperative values but remained significantly lower than preoperative levels (both *p* < 0.001). In infant group, changes in velocity (*p* = 0.118) and gradient (*p* = 0.225) from postoperative to follow-up were not statistically significant. Moderate or severe aortic regurgitation recurred in some patients (Figure 2).

**Figure 3:**
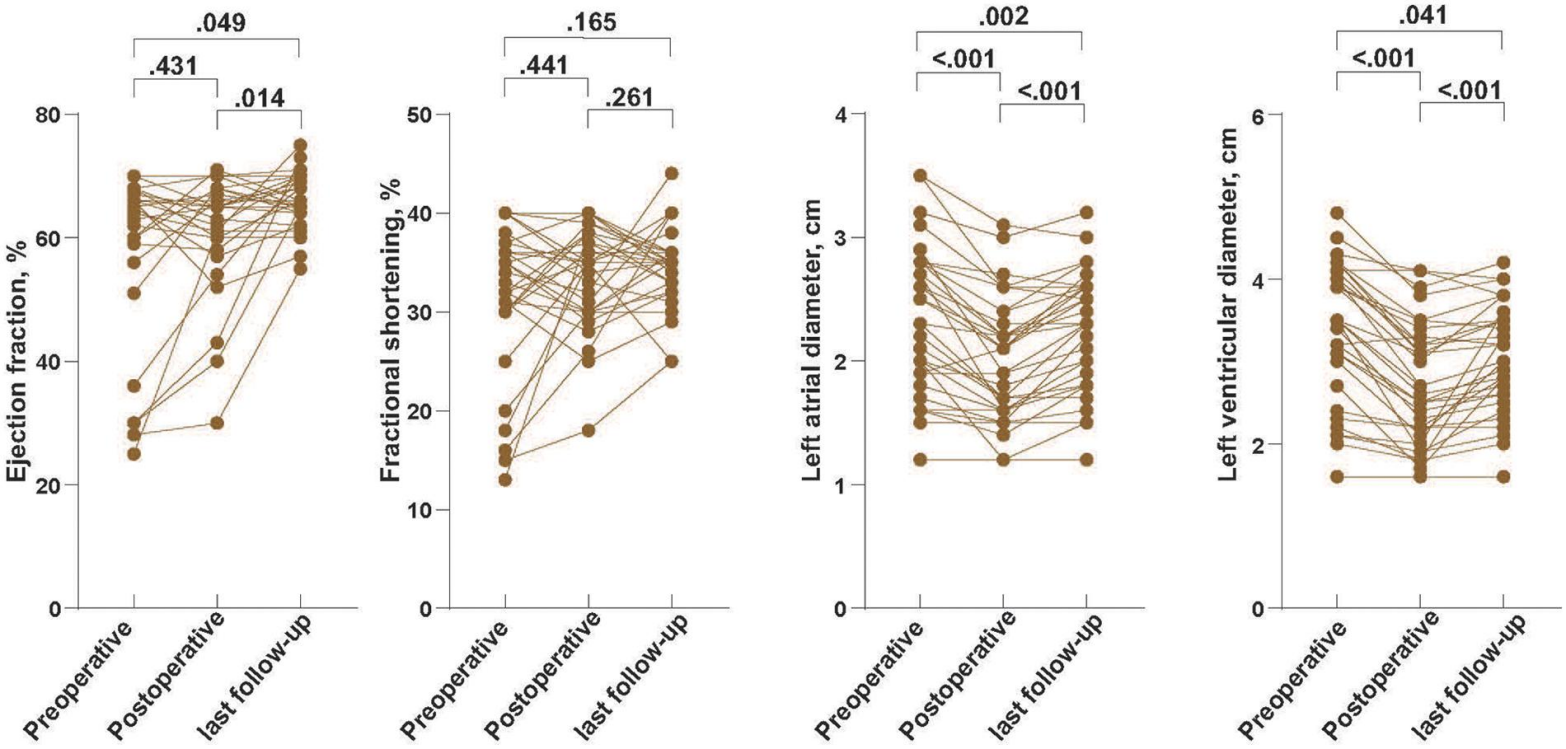
Echocardiographic alterations in left atrial and left ventricular dimensions and systolic function after AVP. Serial transthoracic echocardiographic measurements of left atrial diameter, left ventricular end-diastolic diameter, left ventricular ejection fraction (LVEF), and fractional shortening (FS) in overall cohort. Analyses were performed based on 33 patients with follow-up data.

In the 33 patients with serial TTE data, left ventricular systolic function was generally preserved throughout follow-up (Figure 3). Ejection fraction remained within the normal range at all three time points, with no significant change between preoperative and early postoperative examinations, and a modest but statistically significant increase from early postoperative assessment to last follow-up and from preoperative to last follow-up (*p* = 0.049 and *p* = 0.014, respectively). Fractional shortening likewise remained stable over time, without significant pairwise differences (all *p* ≥ 0.165). In contrast, chamber dimensions showed a consistent reduction after AVP: both left atrial and left ventricular end-diastolic diameters decreased significantly from preoperative to early postoperative assessment and further declined by the time of last follow-up (all *p* ≤ 0.041) (Figure 3).

**Figure 4:**
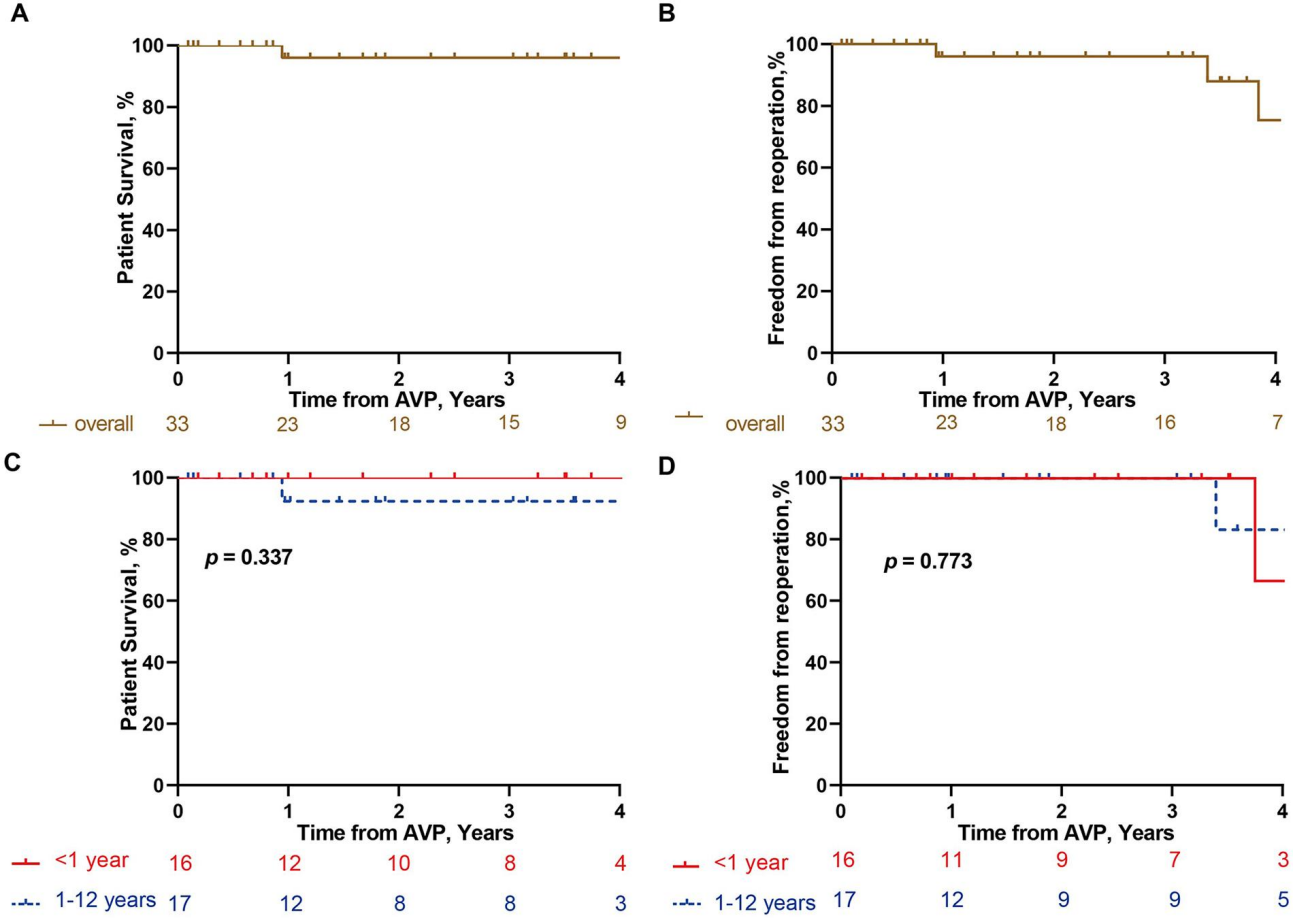
Kaplan–Meier analysis of follow-up outcomes. Kaplan–Meier curves showing overall survival in overall cohort **(A)** and different age groups (≤1 year vs. >1 year) **(C)**, and freedom from reoperation in overall cohort **(B)** and different age groups (≤1 year vs. >1 year) **(D)** Analyses were performed based on 33 patients with follow-up data.

At the four-year follow-up, overall survival was 96% in the overall cohort and 100% in the infant (Table 3, Figure 4A). Freedom from reoperation was 75.4% in the overall cohort and 66.7% among infants. Five patients underwent reoperation during the follow-up period. Indications included recurrent aortic stenosis in two patients (peak gradients of 71 and 110 mmHg) and severe aortic regurgitation in three patients. Two patients underwent Ross procedures, and three underwent mechanical AVR (Table 4, Figure 4B).

**Table 4:** Details of patients who underwent reoperation in follow-up period.

Pt.	1	2	3	4	5
Date of first surgery	2020/11/19	2019/12/26	2019/12/23	2017/8/31	2017/6/19
Age at first surgery	1 month	10 years	2 years	6 months	3 years
Date of reoperation	2024/9/25	2023/5/17	2024/11/29	2024/7/3	2021/7/21
Initial predominant lesion	AS	AS	AR	AS	AS
Peak aortic valve gradient/AR grade at preoperative	66 mmHg/none	81 mmHg/mild	29 mmHg/severe	101 mmHg/mild	93 mmHg/mild
Peak aortic valve gradient/AR grade at postoperative	35 mmHg/none	29 mmHg/mild	27 mmHg/mild	48 mmHg/none	46 mmHg/none
Etiology of Reoperation	AS	AR	AR	AR	AS
Peak aortic valve gradient/AR grade at reoperation	71 mmHg/none	33 mmHg/severe	59 mmHg/severe	40 mmHg/severe	110 mmHg/mild
Type of Reoperation	Ross	mAVR	mAVR	mAVR	Ross

AR, Aortic regurgitation; AS, Aortic stenosis; mAVR, Mechanical aortic valve replacement.

## 4 Discussion

Our findings highlight the safety, technical feasibility, and short-term efficacy of AVP with a pericardial patch in pediatric patients with congenital aortic valve disease, particularly infants. Despite the absence of long-term data, our findings demonstrate favorable hemodynamic improvement and low perioperative morbidity, supporting AVP with pericardium patch as a viable option in selected pediatric cases.

Congenital aortic valve defects remain a major challenge in pediatric cardiac surgery. The use of surgical interventions in children, particularly in neonates and infants, is limited by physiological fragility, small annular size, and rapid somatic growth. While surgical commissurotomy remains the preferred initial intervention for neonatal and infant aortic valve stenosis, postoperative regurgitation is not uncommon (11, 12). Mechanical valves are constrained by the fixed size of prosthetic valves and require lifelong anticoagulation (13), while bioprosthetic valves bioprosthetic valves are prone to calcification and structural degeneration (14). Although the Ross procedure is favored as it accommodates growth, its technical complexity and high early mortality rates in infants limit its applicability (2, 3).

In this context, valve reconstruction with pericardium patch has gained increasing attention. Notably, glutaraldehyde-treated autologous pericardium has demonstrated favorable tensile strength and mid-term pliability in Ozakìs research (8). Surgeons have attempted to employ pericardial patches for leaflet extension to address inadequate leaflet height (15, 16). However, this procedure is technically demanding in ensuring optimal leaflet geometry and coaptation. The Ozaki procedure, which involves full cusp reconstruction and tricuspidization with glutaraldehyde-treated pericardium, has demonstrated promising mid-term outcomes in adult and adolescent cohorts (17). However, its application in infants has limitations including technical complexity, the lack of standardized templates for small aortic roots and growth adaptability. Given these limitations, valve reconstruction techniques that use minimal prosthetic material and involve fewer operative Invasion are more desirable in infants and small children.

In this study, a pericardial patch was applied in patients with congenital aortic valve defects and adequate annular dimensions. The surgical strategy was individualized based on native cusp morphology. For bicuspid aortic valves, we aimed to reconstruct a symmetric tricuspid construction if feasible. For unicuspid valves, a symmetric bicuspidization technique was adopted to achieve favorable hemodynamics. Surgical repair included splitting of fused commissures, thinning of thickened leaflet tissue, and central plication of prolapsed leaflets to achieve uniform cusp height. For commissures reconstruction, we then use a glutaraldehyde-treated autologous pericardial triangular patch to repair leaflet deficiency and reconstruct the interleaflet triangle, following the technique described by Monro and colleagues (7). This approach results in a shorter cross-clamp time, avoids extensive leaflet replacement and minimizes surgical invasiveness, while preserves native cusp and annular geometry. These improvements make the technique suitable for infants with fragile tissues, limited anatomical space, and ongoing growth.

Postoperative TTE showed effective improvement in hemodynamic and no newly developed moderate or severe aortic regurgitation in any patient. Also, left ventricular function therefore appeared to be preserved over the mid-term follow-up, with no echocardiographic evidence of adverse remodeling despite effective relief of pressure and/or volume overload after AVP. Importantly, there were no perioperative deaths or major complications in hospital period. During follow-up, if patients met established surgical criteria, they were advised to be readmitted for further assessment and reoperation if indicated. In this study, one patient experienced late mortality during follow-up due to recurrent aortic stenosis. Although satisfactory early hemodynamic improvement was achieved after AVP, progressive restenosis developed within the first year after surgery and ultimately led to heart failure. This adverse outcome may reflect limitations of early surgical experience and highlights the importance of optimal intraoperative valve reconstruction. Based on observations from subsequent cases in this cohort, the use of a glutaraldehyde-treated autologous pericardial patch appeared effective in preventing early postoperative aortic regurgitation. Adequate commissurotomy and optimization of leaflet mobility may be critical for achieving durable valve function, particularly in patients with relatively preserved native cusps. In addition, in patients with significant ascending aortic dilation, concomitant aortic intervention should be considered to reduce the risk of late adverse events (18). During the follow-up period, four patients required reoperation and achieved satisfactory outcomes. This technique may delay the need for more radical and invasive interventions until a later age. In summary, AVP with pericardium patch appears to meet our expectations for achieving meaningful hemodynamic improvement via a safe and durable repair strategy. This approach is particularly well-suited for infants, providing a bridge to future definitive interventions while accommodating somatic growth.

### 4.1. Limitations

This study has several limitations. First, it represents a single-center, single-surgeon retrospective experience, and the decision to perform AVP with a pericardial patch was based on multidisciplinary clinical judgment, which may introduce selection bias as well as a learning-curve effect. Second, the sample size was relatively small, particularly in key subgroups such as infants and patients requiring reoperation, which limits statistical power and the ability to perform robust subgroup analyses. Third, the follow-up duration was modest, and long-term durability of the repair, especially in relation to somatic growth, could not be fully assessed. In addition, aortic annular dimensions were not systematically measured at all follow-up visits, precluding a detailed evaluation of annular growth over time. Finally, multiple hemodynamic parameters were compared without formal adjustment for multiple testing; therefore, the possibility of type I error inflation cannot be excluded. These limitations underscore the need for larger, multicenter studies with longer follow-up to validate the present findings.

## 5 Conclusion

This retrospective study suggests that AVP with pericardium patch is a safe and effective operation for pediatric patients aged under 12 years with congenital aortic stenosis and regurgitation, offering favorable short-term outcomes. It may be considered a promising treatment approach, particularly for infants.

## Data Availability

The raw data supporting the conclusions of this article will be made available by the authors, without undue reservation.
