# Supplementary material for: Outcomes of aortic valvuloplasty with pericardium patch for congenital aortic stenosis and regurgitation in pediatric patients
Source: Front Cardiovasc Med. 2026 Jan 21;12:1724329. doi: 10.3389/fcvm.2025.1724329 (PMC12868139; doi:10.3389/fcvm.2025.1724329)
Supplement: Supplementary file 1 [file Table1.docx]

Supplementary Material

# Supplementary Tables

**Supplementary Table S1. Preoperative and Postoperative Echocardiogram and CTA Outcomes of Infants.**

|  | Overall  (*n* = 35) | | | Age ≤1 year  (*n* = 17) | | | Age >1 year  (*n* = 18) | | |
| --- | --- | --- | --- | --- | --- | --- | --- | --- | --- |
|  | Preoperative | Postoperative | *p*-value | Preoperative | Postoperative | *p*-value | Preoperative | Postoperative | *p*-value |
| Peak aortic valve velocity, m/s | 4.1 (3.7, 4.6) | 2.9 (2.5, 3.2) | <0.001 | 4.0 (3.8, 4.3) | 2.7 (2.4, 3.0) | <0.001 | 4.3 (3.7, 4.8) | 3.0 (2.8, 3.4) | <0.001 |
| Peak aortic valve gradient, mmHg | 67.0 (53.0, 84.0) | 33.0 (26.2, 40.8) | <0.001 | 63.0 (54.5, 74.5) | 29.0 (24.0, 35.0) | <0.001 | 73.5 (55.8, 90.8) | 36.0 (31.0, 46.0) | <0.001 |
| Ascending aorta diameter, cm | 1.9 (1.4, 2.8) | 1.5 (1.1, 2.4) | < 0.001 | 1.4 (1.1, 1.6) | 1.1 (1.0, 1.5) | 0.052 | 2.7 (2.2, 3.1) | 2.4 (1.9, 2.6) | <0.001 |
| Left atrial diameter, cm | 2.5 (1.9, 2.8) | 2.1 (1.6, 2.3) | < 0.001 | 1.8 (1.6, 2.2) | 1.6 (1.5, 1.8) | 0.019 | 2.8 (2.5, 2.9) | 2.2 (2.1, 2.4) | <0.001 |
| Left ventricular diameter, cm | 3.4 (2.4, 4.0) | 2.6 (2.1, 3.3) | < 0.001 | 2.4 (2.1, 2.9) | 2.1 (1.9, 2.4) | <0.001 | 4.0 (3.6, 4.2) | 3.3 (3.1, 3.6) | <0.001 |
| Interventricular septal thickness, cm | 0.6 (0.5, 0.9) | 0.6 (0.5, 0.8) | 0.586 | 0.5 (0.4, 0.6) | 0.5 (0.5, 0.6) | 0.632 | 0.8 (0.7, 0.9) | 0.8 (0.7, 0.9) | 0.190 |
| Right atrial diameter, cm | 2.2 (1.8, 2.9) | 2.2 (1.7, 2.7) | 0.481 | 1.7 (1.6, 1.8) | 1.7 (1.7, 2.0) | 0.376 | 2.8 (2.6, 3.1) | 2.7 (2.5, 3.2) | 0.548 |
| Right ventricular diameter, cm | 2.2 (1.8, 2.9) | 2.3 (1.7, 2.8) | 0.709 | 1.7 (1.6, 1.9) | 1.7 (1.6, 1.8) | 0.676 | 2.8 (2.5, 3.0) | 2.8 (2.5, 3.0) | 0.864 |
| Pulmonary artery trunk diameter, cm | 1.6 (1.2, 1.9) | 1.4 (1.1, 1.8) | 0.049 | 1.1 (1.1, 1.3) | 1.1 (0.9, 1.2) | 0.010 | 1.9 (1.7, 2.0) | 1.7 (1.6, 1.9) | 0.016 |
| Fractional shortening, % | 35.0 (32.0, 38.0) | 35.0 (30.5, 36.5) | 0.820 | 35.0 (27.5, 36.5) | 35.0 (30.2, 36.0) | 0.734 | 35.0 (33.0, 39.5) | 35.0 (32.0, 37.0) | 0.209 |
| Ejection fraction, % | 65.0 (60.0, 68.0) | 65.0 (60.0, 66.8) | 0.162 | 61.5 (31.5, 67.5) | 63.0 (54.0, 66.0) | 0.225 | 65.0 (62.0, 69.5) | 65.0 (61.0, 67.0) | 0.361 |
| Left ventricular outflow tract velocity, m/s | 0.8 (0.6, 1.0) | 0.8 (0.7, 1.0) | < 0.001 | 0.7 (0.5, 0.8) | 0.8 (0.6, 1.0) | 0.058 | 1.0 (0.8, 1.1) | 0.9 (0.7, 1.0) | 0.608 |
| Aortic regurgitation |  |  | <0.001 |  |  | NA |  |  | <0.001 |
| None | 17 (49) | 16 (46) |  | 12 (71) | 7 (41) |  | 5 (28) | 9 (50) |  |
| Mild | 12 (34) | 19 (54) |  | 4 (24) | 10 (59) |  | 8 (44) | 9 (50) |  |
| Moderate | 1 (3) | 0 (0) |  | 0 (0) | 0 (0) |  | 1 (6) | 0 (0) |  |
| Severe | 5 (14) | 0 (0) |  | 1 (6) | 0 (0) |  | 4 (22) | 0 (0) |  |

Data are presented as the median (IQR) for continuous variables and number (percentage) for categorical variables. Abbreviations: CTA, computed tomography aortography. NA, Not Available.
